# Magnetoimpedance Effect in Cobalt-Based Amorphous Ribbons with an Inhomogeneous Magnetic Structure

**DOI:** 10.3390/s23198283

**Published:** 2023-10-07

**Authors:** Dmitry A. Bukreev, Michael S. Derevyanko, Alexander V. Semirov

**Affiliations:** Department of Physics, Pedagogical Institute, Irkutsk State University, 664003 Irkutsk, Russia; mr.derevyanko@gmail.com (M.S.D.); semirov@mail.ru (A.V.S.)

**Keywords:** magnetoimpedance tomography, magnetoimpedance effect, amorphous magnetically soft alloys, computer simulation, finite element method

## Abstract

The results of a computer simulation and experimental study of the magnetoimpedance effect (MI) in amorphous Co_68.5_Fe_4.0_Si_15.0_B_12.5_ and Co_68.6_Fe_3.9_Mo_3.0_Si_12.0_B_12.5_ ribbons in the ac frequency range from 0.01 to 100 MHz are presented. It was found that the maximum MI value exceeds 200%, which may be of interest in the development of magnetic field sensors. It is also shown that practically significant characteristics of the MI response strongly depend on the ac frequency, which is due to the inhomogeneous distribution of magnetic properties over the ribbon cross section. This distribution was studied using magnetoimpedance tomography based on the analysis of the experimental dependences of the reduced impedance on the ac frequency.

## 1. Introduction

The magnetoimpedance effect (MI) consists of changing the electrical impedance of a ferromagnetic conductor under the action of an external magnetic field [1]. If we confine ourselves to conductors of planar geometry, then MI is most intensively studied in amorphous magnetically soft ribbons based on cobalt and iron [2,3], including those after nanocrystallization [4,5], as well as in thin magnetic films [6,7] and multilayer films [8,9,10]. In this case, the MI is most often studied in the so-called longitudinal configuration, when the alternating current and the external magnetic field are oriented along the same axis [11]. 

The relative change in the impedance in the mentioned objects can reach hundreds of percents when the magnetic field intensity changes by units of A/m. For this reason, the development of highly sensitive magnetic field sensors based on MI is being actively discussed because it can be in demand both in technical [12,13,14] and biomedical applications [15,16,17].

The MI can be clearly explained in terms of the skin effect. The distribution of an alternating electric current (ac) over the cross section of a conductor with magnetic permeability *μ* and specific conductivity *σ* depends on the ac frequency *f* and can be characterized by the thickness of the skin layer [18]:

(1) $$\delta =\sqrt{\frac{1}{\pi f\sigma \mu_{0}\mu }},$$

where *μ*_0_ is the magnetic constant.

Simplistically, it can be assumed that the alternating current is mainly concentrated in the surface layer of the conductor with a thickness equal to the thickness of the skin layer, i.e., the effective cross section of the conductor is determined by *δ*. Thus, it follows from expression (1) that if the magnetic permeability of the conductor changes, then its effective cross section changes. Consequently, the impedance also changes, i.e., MI is observed.

With a uniform distribution of the magnetic permeability over the cross section of a planar conductor with a thickness of 2*b*, its impedance, taking into account the external inductance *L*_ext_, can be represented as follows [18,19]:

(2) $$\dot{Z}=R_{DC}kb\cot kb+j\cdot 2\pi fL_{\mathrm{ext}},$$

where *R*_DC_ is the ribbon resistance to direct current and *k =* (1 − *j*)/*δ*, *j* is an imaginary unit. External inductance is due to the magnetic flux created by the electric current outside the conductor. In the case of a rectangular conductor with length *l*, width *a* and thickness 2*b*, the value of *L*_ext_ can be written as follows [20]:

(3) $$L_{\mathrm{ext}}=\frac{\mu_{0}l}{2\pi }\left(\ln\frac{2l}{2b+a}+\frac{1}{2}\right),$$

Equation (2) shows that the contribution of an external inductance increases with an ac frequency increase. With the usual parameters for samples of cobalt-based ribbons (*a* = 1 mm, 2*b* = 25 μm and *σ* = 600 kS/m), the contribution of the external inductance at an ac frequency of 1 MHz is 10 times less than *R*_DC_, that at the frequency of 10 MHz becomes comparable and that at the frequency of 100 MHz exceeds *R*_DC_ by 10 times. The contribution of the external inductance is often not considered when modeling MI at low ac frequencies [19]. However, at high frequencies, this contribution must be considered.

To date, theoretical concepts have been developed on how to determine the orientation of an easy magnetization axis and the magnitude of magnetic anisotropy on MI of planar magnetically soft conductors [19,21]. The frequency dispersion of the magnetic permeability and different orientations of the magnetization in neighboring magnetic domains were considered in [22]. The MI was also considered in the presence of an inhomogeneous external magnetic field [23]. The theoretical models of MI are of interest in multilayer films, the electrical and magnetic properties of which vary over the cross section [10,24]. A computer simulation using the finite element method also recommended itself in the study of MI response of such objects [8,25,26]. The effect of various coatings on the MI of amorphous magnetically soft ribbons was also studied using a computer simulation [27]. However, the influence of the nonuniform distribution of magnetic properties over the cross section of the ribbons on the MI has not been discussed either theoretically or with the help of computer simulations, although, this issue is relevant, as shown below.

Quenching stresses are irregularly distributed over the thickness of the amorphous ribbon. According to [28,29], the quenching stresses reach their maximum modulus near the surface of the ribbon, while they are minimal in its central part. Since there is no magnetocrystalline anisotropy in amorphous ribbons, the magnetoelastic anisotropy caused by quenching stresses leads to the formation of an inhomogeneous magnetic structure. It was shown when studying amorphous FePC ribbons that the distribution of the magnetic anisotropy over the thickness of the ribbon is inhomogeneous and asymmetric [30]. Its value is minimal near the middle of the ribbon and increases as it approaches the ribbon’s surfaces. This distribution of the magnetic anisotropy correlates with the quenching stress distribution over the ribbons’ cross sections described above.

Another factor affecting the distribution of magnetic properties over the cross section of the ribbon is surface irregularities [29,31,32]. The effect of irregularities on the magnetic anisotropy parameters of an amorphous ribbon is especially strong in the case of zero magnetostriction [32]. Surface irregularities can also cause an increase in the magnetic anisotropy dispersion [31,33], which can strongly affect the MI, especially at high frequencies [34]. Polishing and other modifications of the ribbon surface lead to a significant change in the MI response [35,36].

If the magnetic parameters are not uniformly distributed over the ribbon cross section, then different regions of the ribbon, which may have different magnetic anisotropy parameters, are involved in the formation of the MI response at different ac frequencies due to different skin layer thicknesses. In this case, it is fair to expect that the nature of the MI response depends on the ac frequency. This is also supported by numerous experimental results, for example [37]. Thus, the inhomogeneity of the ribbon magnetic structure must be considered when developing MI sensors. Knowing the distribution of the magnetic properties over the ribbon cross section and how it changes depending on the choice of manufacturing conditions and further heat treatment, it is possible to purposefully tune the characteristics of MI sensors based on amorphous ribbons for a specific task.

To establish the distribution of the magnetic properties over the cross section of amorphous and electrodeposited wires, a method called magnetic impedance tomography (MIT) was proposed [38]. This method is based on the analysis of the impedance dependences on the ac frequency. In this paper, we propose an implementation of this method for amorphous ribbons.

## 2. Samples, Experimental Methods and Computer Simulation

### 2.1. Description of the Samples

The ribbons Co_68.5_Fe_4.0_Si_15.0_B_12.5_ (S0) and Co_68.6_Fe_3.9_Mo_3.0_Si_12.0_B_12.5_ (S1) were prepared by rapid quenching using the Cu weal technique. The nominal widths of the S0 and S1 ribbons were 0.71 mm and 0.78 mm, respectively. The thicknesses of the S0 and S1 ribbons were 24 and 26 μm, respectively. Samples 30 mm long were cut from the original ribbon. Designations of the samples, their geometrical parameters, values of magnetostriction and specific conductivity are given in Table 1.

### 2.2. Experimental Methods

**Figure 1:**
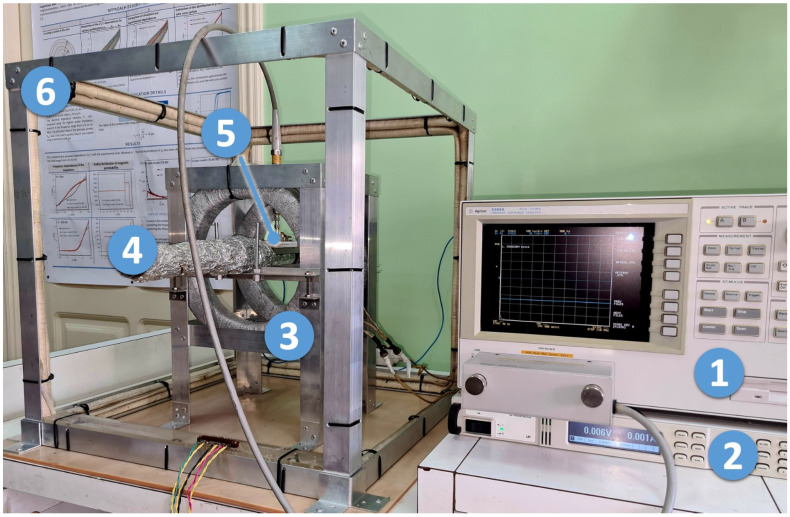
Photograph of the measuring setup. 1—Agilent 4294A impedance analyzer (Keysight Technologies, Santa Rosa, CA, USA); 2—Agilent N6700B (Keysight Technologies, Santa Rosa, CA, USA) power supply for Helmholtz coils; 3—Helmholtz coils; 4—pipe-holder of the measuring cell, which can also be used as part of a heating system (more details in [11]); 5—measuring cell; 6—three pairs of Helmholtz coils to compensate for geomagnetic and effective laboratory fields (coils’ power supplies are not shown).

The modulus of the electrical impedance *Z* was measured using the measuring complex of magnetoimpedance spectroscopy. A measuring setup was developed by the authors of this article. A photograph of the setup is shown in Figure 1; its detailed description is given in [11]. The distance between the contacts of the measuring cell was *l* = 24 mm. The effective value of the alternating current was equal to 1 mA. The alternating current frequency, *f*, varied in the range 0.01–80 MHz. An external magnetic field, *H*, was generated by a pair of Helmholtz coils. Its maximum strength was *H*_max_ = ±12.3 kA/m. The alternating current and the external magnetic field were oriented along the length of the sample.

MI was calculated using the following formula:

(4) $$\Delta Z/Z\left(H\right)=\frac{Z\left(H\right)-Z\left(H_{\max}\right)}{Z\left(H_{\max}\right)}\times 100\%.$$

The magnetic hysteresis loops were obtained using the induction method. The remagnetizing magnetic field oriented along the length of the sample varied with a frequency of 1 kHz, and its amplitude was 1.2 kA/m. 

Saturation magnetostriction, *λ*_S_, was determined using the approach of changing the peak field in the Δ*Z*/*Z*(*H*) dependence under the action of tensile mechanical stresses *γ*. This approach was proposed in [39]. The maximum values of the mechanical stresses, *γ*_max_, were 575 MPa and 480 MPa for the S0 and S1 ribbons, respectively.

### 2.3. Computer Simulation of the MI

**Figure 2:**
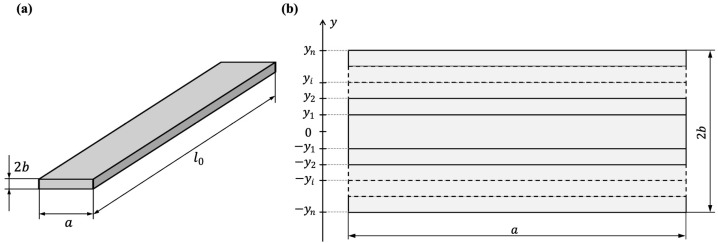
Schematic representation of the ribbon (**a**) and splitting the ribbon model into layers (**b**).

A computer simulation of the MI was performed using the finite element method in Comsol Multiphysics software in the ac frequency range from 0.01 to 80 MHz (license no. 9602434). The ribbon model had a rectangular section 2*b* × *a* and length *l*. The section of the model was divided into a stack of 2*n* − 1 layers of a rectangular section, arranged symmetrically with respect to its middle (Figure 2b). The width and length of each layer were *a* and *l*, respectively. For each layer, the value of the transverse magnetic permeability *μ_i_* was set according to the following system of equations:

(5) $$\{\begin{array}{c} \mu_{1},\;\;if\;\left|y\right|\leq \;|y_{1}|;\\ \mu_{2},\;if\;\left|y_{1}\right|<\left|y\right|\leq \;|y_{2}|;\\ \ldots \\ \mu_{i},\;if\;\left|y_{i-1}\right|<\left|y\right|\leq \;|y_{i}|;\\ \ldots \\ \mu_{n},\;if\;\left|y_{n-1}\right|<\left|y\right|\leq \;|y_{n}|, \end{array}$$

where *y_i_* is the coordinate of the outer boundary of the *i*-th layer. Obviously, |*y_n_*| = *b*. The specific conductivities of the layers were assumed to be the same and equal to *σ* (see Table 1). Models with *n* from 1 to 6 were considered.

**Figure 3:**
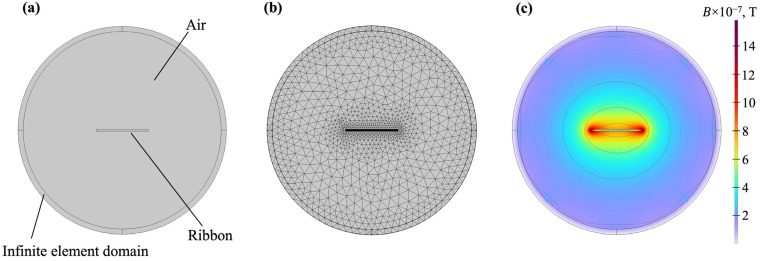
A model for calculating the MI response of the ribbon with the finite element method using Comsol Multiphysics (**a**), fragmentation into a finite element mesh (**b**) and the result of calculating the magnetic induction in the ribbon and the surrounding space (**c**).

To simulate the magnetic flux outside the ribbon in Comsol Multiphysics, the circular section Air with radius 2*a* was used (Figure 3). The magnetic permeability of this region is 1, the permittivity is 1 and the specific conductivity is 0. The closing of the magnetic field lines at infinity was considered using the Infinite Element Domain tool (outer cylindrical layers of the model in Figure 3). It should be noted that, we made sure that the size of finite element mesh elements was less than the thickness of the skin layer. Figure 3c also shows an example of calculating the distribution of magnetic induction in and around the ribbon. 

### 2.4. Implementation of Magnetic Impedance Tomography of Amorphous Ribbons

MIT was used in this work to estimate the distribution of magnetic permeability over the amorphous ribbons’ cross sections. This method was implemented as described below.

1. The distribution of the magnetic permeability was set according to the system of Equation (5) and Table 2. In this case, *μ_i_* varied from 1 to 25,000.
2. Using solutions for the electric and magnetic field obtained with Comsol Multiphysics for various combinations of *μ_i_* values, the dependences of the reduced impedance on the ac frequency *Z*(*f*)/*R*_DC_ were calculated.
3. In the array of the simulated *Z*(*f*)/*R*_DC_ dependences, we found the one that had the smallest absolute deviation from the *Z*(*f*)/*R*_DC_ dependence obtained experimentally.
4. The combination of *μ_i_* values at which the simulated dependence *Z*(*f*)/*R*_DC_ has the smallest deviation from the experimental one is, presumably, an approximation of the actual distribution of the magnetic permeability over the ribbon cross section.

The dependence of the magnetic permeability on the ac frequency can be described using the Landau–Lifshitz–Gilbert equation (see for example [40]). The greater the Gilbert damping parameter *k_G_* included in this equation, the more the magnetic permeability changes with frequency. For amorphous CoFeSiB alloys, the typical *k_G_* value is around 0.03 [41]. With this parameter value, the magnetic permeability module at the frequency of 80 MHz differs from the magnetic permeability module at the frequency of 0.01 MHz by several percent. Therefore, to simplify the modeling in this work, we neglect the frequency dependence of the magnetic permeability modulus. However, it should be noted that the real and especially imaginary component of the magnetic permeability changes with the ac frequency much more significantly than the modulus. Therefore, taking into account magnetic permeability dependence on the ac frequency is strictly necessary when determining its components using MIT. Obviously, for this, it will be necessary to analyze not the impedance modulus, but the resistance and reactance. The determination of the magnetic permeability components using MIT will be discussed in further articles devoted to the development of this method.

## 3. Results and Discussion

### 3.1. Experimental Results

**Figure 4:**
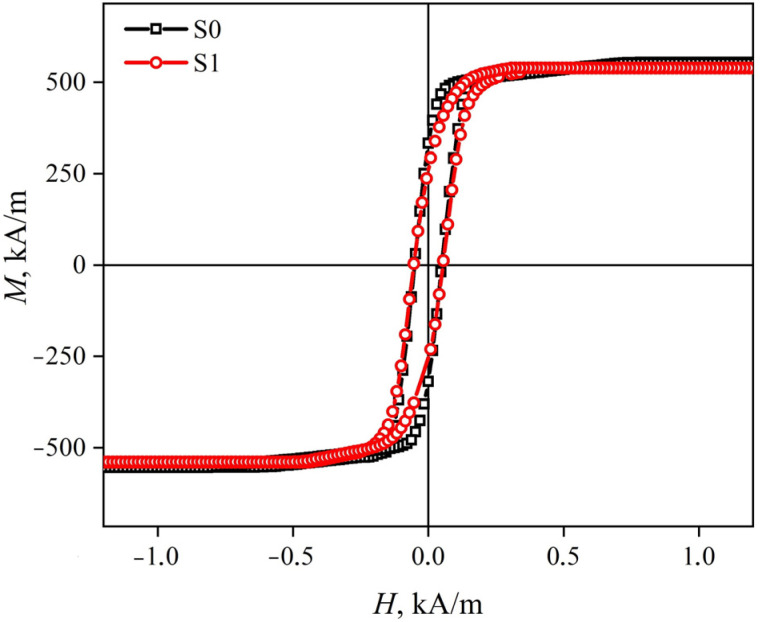
Magnetic hysteresis loops of S0 and S1 ribbons.

The saturation magnetization of both ribbons is about *M*_S_ ≈ 560 kA/m (Figure 4). The coercive force in both cases is about 50 A/m. Thus, according to the magnetic hysteresis loops, the ribbons have pronounced soft magnetic properties.

The saturation magnetostriction, determined as described in Section 2.2, is –0.18⋯10^−7^ and +0.59⋯10^−7^ for samples S0 and S1, respectively. It should be noted that the obtained magnetostriction values are in good agreement with the results obtained for similar ribbons based on the analysis of changes in hysteresis loops under the action of tensile mechanical stresses [42].

**Figure 5:**
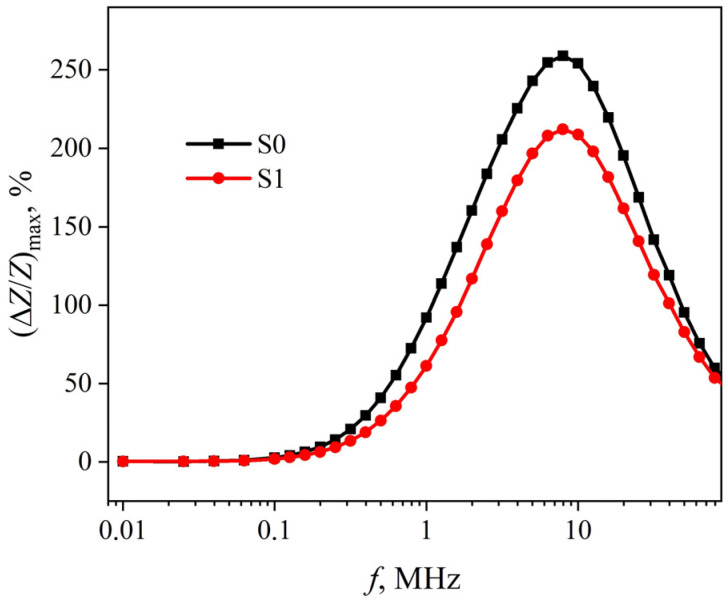
Frequency dependences of the highest MI value (Δ*Z*/*Z*)_max_(*f*) in S0 and S1 ribbons.

The value of (Δ*Z*/*Z*)_max_ corresponds to the peak in the magnetic field dependence of the MI and reaches its maximum value at an ac frequency of about 8 MHz in the case of S1 ribbons (Figure 5). In this case, (Δ*Z*/*Z*)_max_ exceeds 200%, which may be of practical interest. 

In the case of the S0 ribbons, the dependence (Δ*Z*/*Z*)_max_(*f*) has the same characteristics as in the case of S1 ribbons (Figure 5). However, the highest MI value in these ribbons exceeds 250%, which is observed at an ac frequency of about 8 MHz.

**Figure 6:**
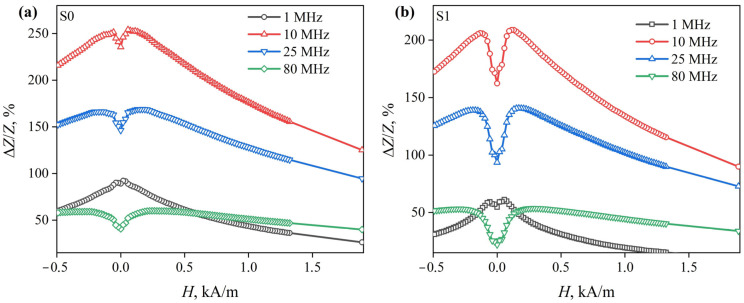
Dependences of the magnetoimpedance effect in the S0 (**a**) and S1 (**b**) ribbons on the external magnetic field strength, obtained at various ac frequencies.

Both in the case of the S1 ribbons and of the S0 ribbons, the MI dependences on the external magnetic field strength Δ*Z*/*Z*(*H*) in the entire ac frequency range have two peaks. This is expressed as the stronger the Δ*Z*/*Z*(*H*), the higher the *f*. In addition, an increase in the magnetic field strength *H*_p_ is observed with an increase in the frequency of the alternating current. For S1 ribbons, the minimum value of the peak field is *H*_p1_ ≈ 60 A/m and the maximum value is *H*_p2_ ≈ 320 A/m (Figure 6b). In turn, for S0 ribbons, these values are approximately 20 and 260 A/m, respectively (Figure 6a). The described changes in *H*_p_ with ac frequency increasing, as well as an increase in the increasing portion of the Δ*Z*/*Z*(*H*) dependence may be associated with the inhomogeneous distribution of the magnetic permeability over the cross section of the S0 and S1 ribbons [38].

### 3.2. MIT Results

**Figure 7:**
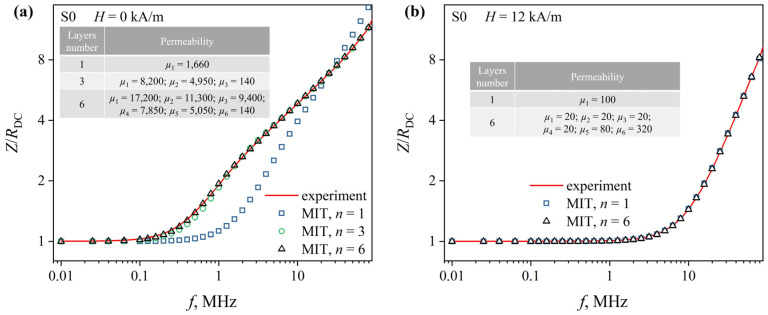
Frequency dependences of the reduced impedance *Z*(*f*)/*R*_DC_ of the S0 ribbons. The dependences were obtained in magnetic fields of 0 (**a**) and 12 kA/m (**b**). Lines are experimental dependences; markers—dependencies restored using MIT. The tables show the magnetic permeability values reconstructed using MIT.

**Figure 8:**
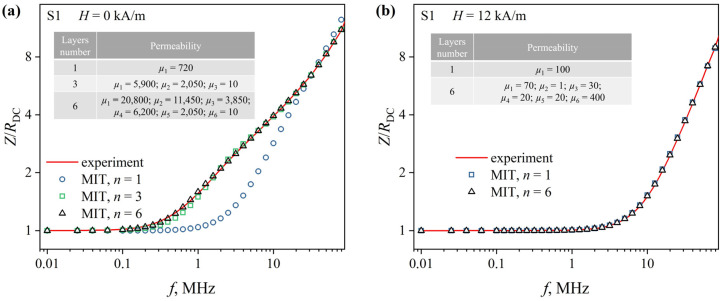
Frequency dependences of the reduced impedance *Z*(*f*)/*R*_DC_ of the S1 ribbons. The dependences were obtained in magnetic fields of 0 (**a**) and 12 kA/m (**b**). Lines are experimental dependences; markers—dependencies restored using MIT. The tables show the magnetic permeability values reconstructed using MIT.

Details of the magnetic permeability distribution over the ribbon cross section were restored using MIT (see Section 2.4) based on the analysis of the experimental *Z*(*f*)/*R*_DC_ dependences (Figure 7 and Figure 8, solid lines). These dependencies consist of two sections: an almost horizontal section *Z*/*R*_DC_ ≈ 1, which at a certain alternating current frequency *f*_0_, smoothly transforms into an increasing section. It should be noted that there are features such as kinks and changes in the angle of inclination in low magnetic fields in the increasing section of the *Z*(*f*)/*R*_DC_ dependence. It may indicate a non-uniform distribution of the magnetic permeability over the cross section of the ribbon [38]. In magnetic fields close to *H*_max_, the increasing section is almost a straight line, indicating a distribution of magnetic permeability that is close to uniform.

The *Z*(*f*)/*R*_DC_ dependences reconstructed from the MIT results at *n* = 1 (uniform distribution of the magnetic permeability over the ribbon cross section) differ significantly from the experimental ones obtained in low magnetic fields (Figure 7a and Figure 8a, markers). The relative deviation for some ac frequencies exceeds 80% and confirms the conclusion about the inhomogeneous magnetic structure of the ribbons. An increase in *n* in the model leads to a decrease in the deviation of the reconstructed *Z*(*f*)/*R*_DC_ dependences from the experimental ones. Thus, at *n* = 3, the *Z*(*f*)/*R*_DC_ dependences reconstructed using MIT deviate from the experimental ones by no more than 6%. Increasing the number of layers to six made it possible to ensure that the deviation did not exceed 3% over the entire studied range of the magnetic fields.

At the same time, at *H*_max_, the *Z*(*f*)/*R*_DC_ dependences, constructed from the MIT results, even at *n* = 1, deviate from the experimental ones by no more than 3% (Figure 7b and Figure 8b markers). This confirms the conclusion that with external magnetic field strength increasing, the distribution of the magnetic permeability over the ribbons cross section becomes more uniform. When using the model with *n* = 6, the deviation of the reconstructed and experimental dependencies does not exceed 1%.

It should also be noted that the deviation of the simulated and experimental dependences may be due to the fact that the frequency dispersion of the magnetic permeability, including the dispersion of the magnetic permeability associated with the motion of domain walls, is not taken into account. Moreover, symmetrical models were used for MIT, while the distribution of magnetic permeability can be asymmetric due to the asymmetric distribution of hardening stresses and the different state of the contact and free surfaces of the ribbon [28,29,30,31,32,33].

**Figure 9:**
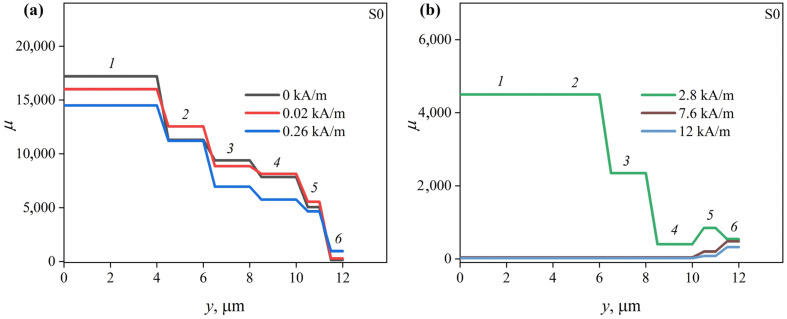
Distributions of the magnetic permeability over the S0 ribbon cross section in the magnetic fields with strengths (**a**) 0, 0.02 and 0.26 kA/m and (**b**) 2.80, 7.6 and 12.4 kA/m.

**Figure 10:**
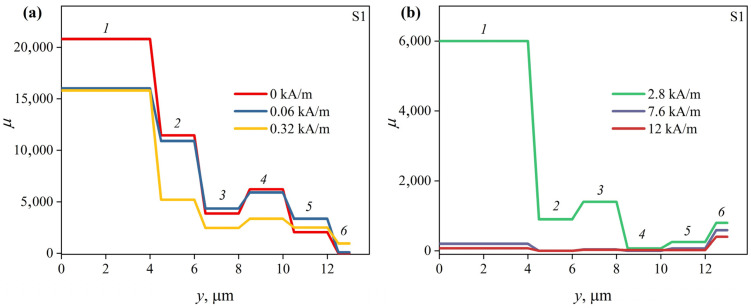
Distributions of magnetic permeability over the S1 ribbon cross section in magnetic fields with strengths (**a**) 0, 0.06 and 0.32 kA/m and (**b**) 2.80, 7.6 and 12.4 kA/m.

Magnetic permeability distributions reconstructed using MIT for *n* = 6 are shown in Figure 9 for the S0 ribbon and in Figure 10 for the S1 ribbon. 

According to the MIT results, the permeability of the S0 ribbon surface layer that is 1 µm thick is about 140 in the absence of the external magnetic field (Figure 9). The permeability of the next layer is much higher—more than 4000. With further advancement in depth, the magnetic permeability gradually increases, reaching 17,000 in the central part of the ribbon.

The permeability of the surface layer of the S1 ribbon is also low—about 10 (Figure 10). However, the details of the magnetic permeability distribution in the inner regions of the S1 ribbon are somewhat different from those of the S0 ribbon. Thus, at *H* = 0, the magnetic permeability first increases from 2000 to 6200, then decreases to 3800 and then increases again when moving deep into the ribbon from layer 5 to layer 2 (see Table 2). The magnetic permeability of the central layer exceeds 20,000. Note that internal quenching stresses at some distance from the ribbon surface can change their sign, passing through 0 [28,29]. In this region, the magnetoelastic energy 3*λ*_s_*γ_in_*/2 (*γ_in_* is the internal quenching stress) can also be near zero. This, apparently, determines the higher magnetic permeability in layer 4 compared to that in neighboring layers.

Next, consider how the magnetic permeability changes with a change in the external magnetic field.

As *H* increases, the magnetic permeability of the outer layer of the S0 ribbon first increases, reaching a maximum value of about 950 at *H* = *H*_p2_, and then decreases. It reaches 300 at *H*_max_ (Figure 9). The magnetic permeabilities of layers 4–5, as well as the permeability of layer 2, reach their maximum values in lower fields, at *H* = *H*_p1_. The magnetic permeabilities of the remaining layers decrease with increasing external magnetic field strength. Note that the inner layers, in contrast to the surface ones, are close to saturation at *H*_max_. Their magnetic permeabilities are about 1.

In the case of ribbon S1, an increase in the magnetic field strength from 0 to *H*_p1_ leads to an increase in the magnetic permeability of layers 3, 5 and 6. The permeability of layer 4 practically does not change in this case. The magnetic permeability of layer 5 increases most noticeably. The magnetic permeability, as in the case of S0 ribbons, decreases in the entire ribbon with the subsequent increase in *H*, except for the surface layer. Its permeability continues to increase, reaching a value of about 950 and then decreasing to about 400 at *H*_max_.

Probably, the increase in the MI at low frequencies is mainly due to an increase in the magnetic permeability of the inner regions of the ribbon both in the case of the S1 ribbon and in the case of the S0 ribbon. The nature of the Δ*Z*/*Z*(*H*) dependence at high frequencies is mainly determined by the magnetic field dependence of the magnetic permeability surface layer.

The peculiarities of the change in the magnetic permeability of the surface layers of both ribbons indicates a predominantly transverse orientation of the EMA in them. At the same time, the maximum values of these permeabilities are small. This is probably due to the significant anisotropy dispersion caused by non-uniform relief of the ribbon. Also, the magnetic permeabilities of the surface layers of the ribbons are far from saturation, even at *H*_max_, due to the anisotropy dispersion. This, along with the high contribution of the external inductance 2*πfL*_ext_ (see expression (2)) limits the maximum MI at high ac frequencies (Figure 5), which must be taken into account when developing MI sensors.

The magnetic anisotropy of the ribbon inner regions is predominantly longitudinal. This is indicated by high values of the magnetic permeability and its decrease with increasing *H*. An increase in the permeability of some inner layers with a change in the magnetic field from 0 to *H*_p1_ is presumably associated with a decrease in the magnetic anisotropy dispersion. Based on this, it can be assumed that quenching stresses do not have a decisive effect on the orientation of the EMA. Otherwise, the orientation of the EMA in the inner and outer regions of the S0 and S1 ribbons would be different since the signs of their magnetostriction constants are different. It is likely that the EMA orientation is strongly affected by the anisotropy of the ribbons’ shape. However, the energy of magnetoelastic anisotropy can affect the values of magnetic permeability due to the quenching stresses. The magnetoelastic energy 3*λ*_s_*γ_in_*/2 of the S0 ribbon is smaller, since its magnetostriction is smaller in modulus. This leads to the fact that the magnetic permeability of the inner region of the S0 ribbon is higher than that of the S1 ribbon. According to the literature [28,29], the quenching stresses in the central part are very low, which seems to be responsible for the high magnetic permeability of both ribbons.

The practical significance of MIT deserves special attention. As is known, amorphous soft magnetic ribbons obtained by rapid quenching from a melt are characterized by a noticeable scatter of functional characteristics even within the same batch. Despite this, a minimum scatter of metrological characteristics must be ensured when manufacturing devices using these materials, especially MI magnetic field sensors. This can be achieved by selecting pieces of ribbons with similar distributions of magnetic characteristics using MIT. In addition, MIT may be in demand when monitoring changes in the magnetic structure during heat treatment, which is often used to improve the functional properties of amorphous ribbons [43,44].

## 4. Conclusions

Thus, based on the analysis of the experimental Δ*Z*/*Z*(*H*) and *Z*(*f*)/*R*_DC_ dependences, it was shown that Co_68.5_Fe_4.0_Si_15.0_B_12.5_ (S0) amorphous ribbons with magnetostriction *λ*_s_ ≈ –0.18 · 10^−7^ and Co_68.6_Fe_3.9_Mo_3.0_Si_12.0_B_12.5_ (S1) with magnetostriction *λ*_s_ ≈ +0.59 · 10^−7^ have an inhomogeneous magnetic structure. Details of the distribution of magnetic permeability over the ribbons’ cross section were reconstructed using MIT based on an analysis of the experimental *Z*(*f*)/*R*_DC_ dependences obtained in the ac frequency range from 0.01 to 80 MHz in magnetic fields of various strengths.

According to the MIT, the magnetic permeability distribution over the cross section of both ribbons is nonuniform. The surface layers of both ribbons have predominantly transverse magnetic anisotropy. In this case, the low values of their magnetic permeability indicate a significant dispersion of the magnetic anisotropy associated with the surface topography. The magnetic permeability of the inner layers of both ribbons is much higher than that of the surface ones, and the nature of its change in the magnetic field indicates that the magnetic anisotropy of the inner layers is predominantly longitudinal.

The influence of magnetostriction is manifested in the fact that the magnetic permeability of the inner layers of the Co_68.5_Fe_4_Si_15.0_B_12.5_ ribbon is generally higher than that of the Co_68.6_Fe_3.9_Mo_3.0_Si_12.0_B_12.5_ ribbon, since its magnetostriction is lower in the modulus, respectively, and the magnetoelastic energy associated with quenching stresses is less.

The distribution of the magnetic properties over the cross section of amorphous magnetically soft ribbons, reconstructed using MIT, must be taken into account when choosing the optimal operating modes for MI sensors. It should also be noted that the MIT can be in demand in the manufacture of devices based on amorphous ribbons to ensure the minimum spread of their characteristics.

## Figures and Tables

**Table 1:** Sample parameters for experimental study.

Sample	Composition	Length *l*_0_, mm	Width *a*, mm	Thickness 2*b*, µm	Magnetostriction Sign	Specific Conductivity, kS/m
S0	Co_68.5_Fe_4.0_Si_15.0_B_12.5_	30	0.71	24	−	615
S1	Co_68.6_Fe_3.9_Mo_3.0_Si_12.0_B_12.5_	30	0.78	26	+	618

**Table 2:** Coordinates of the layers of ribbon models S0 and S1 when performing MIT.

Sample	Layer Boundary Coordinates, µm					
	*y*_1_	*y*_2_	*y*_3_	*y*_4_	*y*_5_	*y*_6_
S0	4	6	8	10	11	12
S1	4	6	8	10	12	13

## Data Availability

The data are available from the corresponding author upon reasonable request.
